# Environmental exposure assessment in the international prospective study of Chronic Kidney Disease of UnceRtain Etiology (CKDu) in Agricultural Communities (CURE) research consortium: Design and protocol development☆

**DOI:** 10.1016/j.scitotenv.2025.179642

**Published:** 2025-05-22

**Authors:** Marvin González-Quiroz, Anna Aceituno, Shuchi Anand, Alexander van Geen, Lawrence S. Engel, Emmanuel Jarquin, Clemens Ruepert, Nicole Villegas-González, Mariela Arias-Hidalgo, Nora Franceschini, Daylin Anchía-Pastrán, Karla Solano-Diaz, Andrea Corrales-Vargas, Jennifer Crowe, Idalina Cubilla-Batista, Hildaura Acosta, Adriana Mike, Carolina Guzmán-Quilo, Aurora Aragón, Indiana López-Bonilla, Peter Rohloff, Madeleine K. Scammell, Ramón Garcia-Trabanino, Juan Amador Velázquez, Daniel R. Brooks, Sumit Mohan, Jai Radhakrishnan, Balaji Gummidi, Vivekanand Jha, Whitney Collado, Vivek Bhalla, David J. Friedman, Sushrut S. Waikar, Karen Kesler, Lillian Trochinski, P. Lee Ferguson, Patrick J. Parsons, Heileen Hsu-Kim, Carin Huset, Susan Summer Jenkins, Susan R. Mendley, Jill F. Lebov, Bonnie R. Joubert, Ana Navas-Acien

**Affiliations:** a Department of Environmental and Occupational Health, UT School of Public Health San Antonio, The University of Texas Health Science Center at San Antonio, San Antonio, TX, USA; b Centre for Kidney and Bladder Health, University College London, London, UK; c RTI International, 3040 E. Cornwallis Rd., Research Triangle Park, NC 27709-2194, USA; d Stanford University School of Medicine, Medicine, Division of Nephrology, 780 Welch Road, Suite 106, Palo Alto, CA 94304, USA; e Geochemistry, Lamont-Doherty Earth Observatory (LDEO), Columbia Climate School, New York, NY, USA; f University of North Carolina, Department of Epidemiology, Gillings School of Global Public Health, Chapel Hill, NC, USA; g Agency for Agricultural Development and Health (AGDYSA), Col. Las Rosas, P. Las Margaritas 15, San Salvador, San Salvador, SV 01101, El Salvador; h Universidad Nacional de Costa Rica, Regional Institute for Studies on Toxic Substances, Heredia, Heredia CR 86-3000, Costa Rica; i Escuela de Tecnologías en Salud, Universidad de Costa Rica, Sede de Guanacaste, Liberia 315000, Costa Rica; j Escuela de Medicina, Universidad de Costa Rica, Sede Rodrigo Facio, 11501-2060 San José, Costa Rica; k University of North Carolina, Epidemiology, School of Public Health, 137 E. Franklin Street, suite 306 CB#8050, Chapel Hill, NC 27514-3628, USA; l Hospital Regional Dr. Rafael Estevez, Avenida Alejandro Tapia Final Aguadulce, Coclé, Panama; m Universidad de Panamá, Vicerrectoría de Investigación y Postgrado, Facultad de Medicina, Centro de Investigación e Información de Medicamentos y Tóxicos (CIIMET), Panamá, Panama; n Universidad de Panamá, Centro de Investigación e Información de Medicamentos y Tóxicos (CIIMET), Panamá, Panama; o Universidad de San Carlos de Guatemala, Departamento de Toxicología, Facultad de Ciencias Químicas y Farmacia, 3a calle 6-47 zona 1, ciudad de Guatemala 01001, Guatemala; p Wuqu’ Kawoq, 2da Avenida 3-48 Barrio Pacatabaj, Tecpán, Chimaltenango, GT 04006, Guatemala; q Wuqu’ Kawoq, 2da Avenida 3-48, Barrio Pacatabaj, Tecpán, Chimaltenango 04006, Guatemala; r Boston University School of Public Health, Department of Environmental Health, 715 Albany St., Boston, MA 02118, USA; s Centro de Hemodiálisis, C. Gabriela Mistral 211, San Salvador, San Salvador, SV 01101, El Salvador; t Boston University School of Public Health, Department of Epidemiology, Boston, MA, USA; u Boston University, 715 Albany Street, Department of Epidemiology, Boston, MA 02215-1300, USA; v Columbia University Medical Center, Division of Nephrology, Department of Medicine, and Department of Epidemiology, 622 W 168th St PH4-124, New York, NY 10032, USA; w George Institute for Global Health, UNSW, New Delhi, India; x Prasanna School of Public Health, Manipal Academy of Higher Education, Manipal, India; y School of Public Health, Imperial College, London, UK; z Columbia University Mailman School of Public Health, Department of Environmental Health Sciences, New York, NY, USA; aa Stanford University School of Medicine, Medicine, Division of Nephrology, 3180 Porter Drive, Stanford, CA 94305, USA; ab Beth Israel Deaconess Medical Center, Renal Division, 330 Brookline Ave, RN 227B, Boston, MA 02215, USA; ac Section of Nephrology, Boston Medical Center and Boston University Chobanian & Avedisian School of Medicine, 650 Albany Street, EBRC5, Boston, MA 02118, USA; ad RTI International, P O Box 12194, 3040 Cornwallis Rd., Research Triangle Park, NC 27709, USA; ae Department of Civil and Environmental Engineering, Duke University, Durham, NC 27708, USA; af Division of Environmental Health Sciences, Wadsworth Center, New York State Department of Health, Empire State Plaza, Albany, NY 12237, USA; ag Department of Environmental Health Sciences, College of Integrated Health Sciences, University at Albany, 1 University Pl, Rensselaer, NY 12144, USA; ah Duke University, Department of Civil & Environmental Engineering, Box 90287, Durham, NC 27708, USA; ai Public Health Laboratory, Minnesota Department of Health, Saint Paul, MN 55101, USA; aj Department of Nutrition, Gillings School of Global Public Health, USA; ak Department of Pharmacology, UNC School of Medicine, University of North Carolina at Chapel Hill, USA; al National Institute of Diabetes and Digestive and Kidney Diseases, Division of Kidney, Urologic and Hematologic Disease, 2 Democracy Plaza, 6707 Democracy Blvd, Bethesda, MD 20892-5458, USA; am National Institute of Environmental Health Sciences, Division of Extramural Research and Training, Durham, NC, USA

**Keywords:** Chronic kidney disease, CKDu, Research consortium, Environmental exposures, Metals, Trace elements, Pesticides, Polycyclic aromatic hydrocarbons

## Abstract

**Background:**

Chronic kidney disease of unknown etiology (CKDu) is a major health concern among outdoor manual workers in rural Central America and South Asia. The CURE study is a prospective longitudinal cohort designed to investigate CKDu’s environmental risk factors through standardized exposure assessments, questionnaires, and biological and environmental sample collection.

**Methods:**

This manuscript details the development of a standardized exposure assessment protocol within the CKDu CURE Consortium. The study recruits adults (18–45 years) from CKDu hotspots across five countries, ensuring proportional representation across three eGFR categories (20–59, 60–89, ≥90 mL/min/1.73m^2^) and excluding participants with diabetes or other known CKD causes. The CURE study is committed to promptly returning clinically and environmentally relevant test results to participants after analysis.

**Results:**

Exposure assessment includes demographics, healthcare access, nephrotoxic medications, infectious pathogens, and agricultural work conditions (e.g., heat stress, hydration, diet, agrochemicals, toxicants). Biological and environmental samples (water, dust, soil, wristbands) are collected considering seasonal variations. Omics analyses, including metabolomics, will investigate environmental and biological interactions. Statistical analyses will assess relationships between exposures and CKDu onset or progression, incorporating environmental mixture analyses.

**Conclusion:**

CURE will provide critical insights into CKDu risk factors, supporting future research and prevention strategies. A comprehensive exposure assessment will enhance understanding of environmental contributors, guiding interventions at individual, workplace, and community levels to mitigate CKDu’s impact.

## GRAPHICAL ABSTRACT

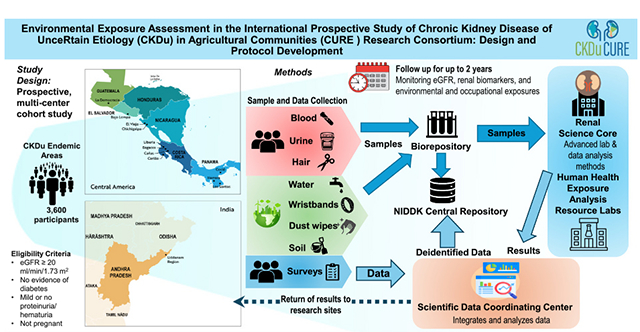

## 1. Introduction

Chronic kidney disease of uncertain etiology (CKDu) has emerged as a major public health problem in hotspot regions in rural communities in Central America and South Asia (Jayasumana et al., 2017; Johnson et al., 2019). CKDu is defined by a presence of a persistently reduced glomerular filtration rate (eGFR) and/or the absence of significant proteinuria or hematuria in individuals without conventional chronic kidney disease (CKD) risk factors such as diabetes and hypertension, or other known kidney disease (Pan American Health Organization, 2017; Crowe et al., 2020). Histologically, CKDu is commonly characterized by chronic tubulointerstitial nephritis, with varying degrees of tubular atrophy, and interstitial fibrosis (Wijkstrom et al., 2017; Wijkstrom et al., 2021). Epidemiologically, the disease disproportionately affects young to middle-aged male agricultural workers in low-resource areas, although there is variability in the presentation (Gonzalez-Quiroz et al., 2024; Rutter et al., 2025). The condition is believed to have a multifactorial etiology involving environmental, occupational, and potentially genetic contributors (Hall et al., 2023; Oomatia et al., 2025; Raines and Rodriguez Garcia, 2024; Stem et al., 2024; Keogh et al., 2022; Rogers et al., 2024; Smpokou et al., 2019; De Broe and Vervaet, 2020; Vervaet et al., 2020). In CKDu-endemic areas, residents are exposed to a complex mixture of environmental hazards including pesticides and other agrochemicals, contaminant metals, and other toxicants in drinking water, dust, soil, and air. Additional hypotheses for CKDu include heat stress in the setting of physically strenuous work, nephrotoxic medications, infectious pathogens, and genetic predisposition (Johnson et al., 2019; Garcia- Trabanino et al., 2015; Holliday Jr. et al., 2022; Sanchez Polo et al., 2020; Valcke et al., 2017; Gonzalez-Quiroz et al., 2018). Prior studies have yielded useful candidate hypotheses, but the incomplete collection of environmental exposures and limited longitudinal follow-up have insufficiently characterized the etiopathogenesis of CKDu and the primary leading factor or factors that contribute to the development of CKDu are unknown (Caplin et al., 2017; Gonzalez-Quiroz et al., 2019; Ulrich et al., 2023).

The Chronic Kidney Disease of Uncertain Etiology in Agricultural Communities (CURE) Research Consortium was established to investigate the development and progression of CKDu and identify relevant etiological factors to facilitate the development of potential therapeutic targets and public health interventions in the affected regions. The CURE Consortium is structured by a network that includes (i) the Scientific Data Coordinating Center (SDCC), (ii) the Renal Science Core (RSC), (iii) Human Health Exposure Analysis Resource (HHEAR) laboratories, (iv) seven Field Epidemiology Sites (research sites/sites - FES) across six countries, and (v) three Institutes of the National Institutes of Health (NIH) — the National Institute of Diabetes and Digestive and Kidney Diseases (NIDDK), the National Institute of Environmental Health Sciences (NIEHS), and the Fogarty International Center (FIC). The CURE study protocol includes the collection of demographics, clinical information, biospecimens, and environmental samples, and longitudinal follow up that aims to: (i) identify risk factors of decline in kidney function, (ii) better characterize the clinical phenotype(s) of individuals with CKDu and differentiate them from other forms of CKD, (iii) employ advanced laboratory and data analysis methods to conduct discovery science related to risk factors, biological markers, and causal mechanisms; and (iv) establish a biorepository for future research. These goals will be accomplished with the active engagement of affected communities and local institutions (Lebov et al., 2025).

The need to test multiple hypotheses including (i) the synergistic effect of environmental mixtures (metals, pesticides, and other nephrotoxic agents) in increasing CKDu risk and accelerating renal function decline beyond the impact of individual exposures, (ii) the interaction between genetic susceptibility and occupational/environmental exposures in driving kidney function decline and CKDu progression over time, and (iii) the potential for dynamic changes in exposure, epigenetic, and metabolomic markers preceding kidney dysfunction, enabling the identification of early CKDu indicators—led to the development of the CURE consortium’s comprehensive exposure assessment protocol. This protocol integrates a multi-faceted evaluation of environmental and occupational risk factors through structured questionnaires, biological sample collection, and the collection of environmental samples (water, dust wipes, soil, and silicone wristbands). This manuscript describes the development and implementation of this comprehensive and standardized exposure assessment protocol following a multi-disciplinary approach as a key strategy to achieve the goals of the CKDu CURE Consortium.

## 2. Study population

The CURE study is a prospective, longitudinal study conducted in CKDu hotspot communities across Costa Rica, El Salvador, Guatemala, Nicaragua, and Panama in Central America, as well as in the Uddanam region of Andhra Pradesh, India. The study aims to enroll a total of 3600 individuals aged 18–45 years (2400 across 5 sites in Central America and 1200 in India). Participants are eligible if, at the time of screening, they have estimated glomerular filtration rate (eGFR) ≥20 mL/min/ 1.73m^2^ as determined locally, no evidence of diabetes (self-report); and hemoglobin A1c (HbA1c) level < 6.5 %, or non-fasting glucose levels below 200 mg/dL; no other known causes of CKD; two kidneys, no kidney stone operations (self-report); evidence of no or limited proteinuria or hematuria; no diagnosis of cancer or treatment with chemotherapy for cancer or rheumatoid arthritis in the past three years; are not pregnant, and have been living in the area for at least one year with no plans to move away. The study aims to enroll participants proportionally across three eGFR categories —CKDu cases (eGFR 20–59 mL/min/1.73m^2^), mildly decreased eGFR (60–89 mL/min/1.73m^2^), and normal eGFR (≥90 mL/min/1.73m^2^). Target enrollment includes ≥80 % men in Central America, and ≥ 60 % men in India within each eGFR stratum, reflecting differences in sex distribution of CKDu prevalence in different locations. Further details on the clinical/study protocol are provided in Lebov et al. (2025) (Lebov et al., 2025).

## 3. Consensus-building framework for exposure assessment in CURE

**Fig. 1.**
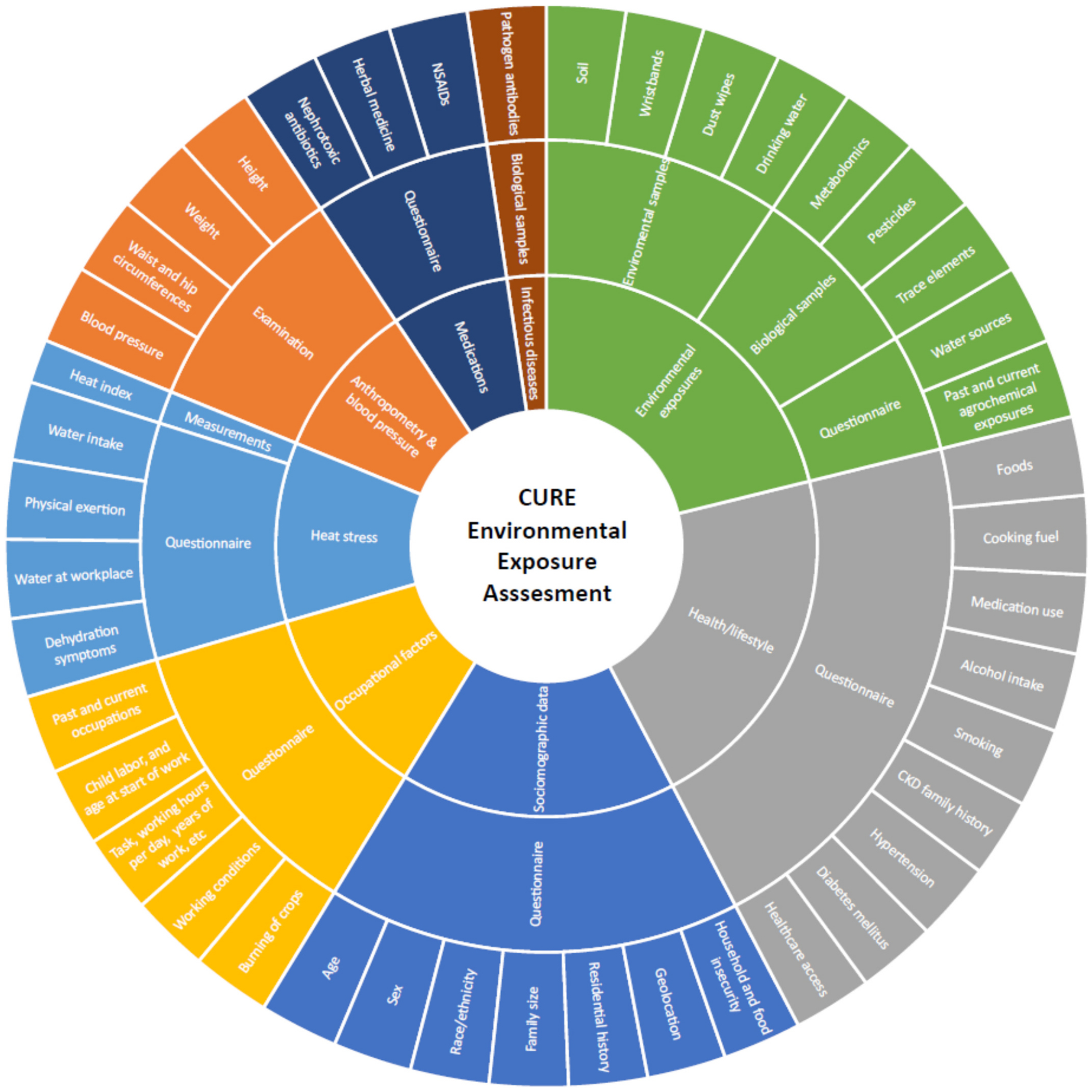
Prioritized exposures to evaluate in the CURE study.

CURE study participants are examined at 3-month intervals between visits 1 and 2, and at 8-month intervals between subsequent visits. Visits include clinical assessment, a structured interview, the collection of biological and environmental samples, and follow-up interviews, covering each country’s rainy and dry seasons. To develop a robust and comprehensive protocol for exposure characterization for participants, >60 investigators from all consortium groups (SDCC, FES, RSC, NIH, and HHEAR) with diverse areas of expertise (e.g., nephrology, physiology, epidemiology, toxicology, environmental sciences, occupational health, pathology, biochemistry, genomics and other -omics, and biostatistics) met regularly to develop the exposure assessment procedures and data collection instruments for CURE. The investigators first identified broad categories of interest, including sociodemographic data, clinical data, medications, occupational conditions (e.g., physical exertion, hydration, and heat exposures), heat stress symptoms, other environmental exposures (e.g., agrochemicals, metals, and other pollutants), infectious pathogens, diet/nutrition, and other lifestyle factors that could affect the kidney function of the study participants. For each of these broad categories, investigators compiled a detailed list of exposures, and each exposure was assesed in terms of its biological plausibility in contributing to CKDu. A consensus-building process was used to select the most plausible factors to include in the exposure assessment protocol. This process leveraged the experience of the multidisciplinary team and considered the existing epidemiological literature, laboratory studies, as well as cultural and community considerations specific to the research sites. For each environmental factor, one or more assessment methods (questionnaire, examination, observation of the home/setting by environmental sampling teams, environmental and or biological sampling, and laboratory analysis) were determined. The investigators also discussed the importance of capturing exposure intensity, duration, and timing (e.g., by measuring exposures at baseline and at repeated time points), recognizing that chronic, low-level exposures can have significant cumulative impacts on kidney health, as well as acute exposures in a specific time window. Exposures were prioritized as low, medium, and high for inclusion in the final study protocol. Prioritization was based on the strength of the prior evidence, prevalence in affected areas, team experience, and potential information gained vs. cost and participant burden. Iterative rounds of discussion helped refine priorities until consensus was reached. Fig. 1 presents the final prioritized list and measurement method of the exposures included, which was approved by the overarching CURE Steering Committee following presentation and discussion with an external Observational Studies Monitoring Board (OSMB).

After the exposures of interest were determined, two sub-groups were formed to operationalize exposure assessment into specific protocols, including standard operating procedures for biological and environmental sample collection and processing for anticipated laboratory analyses. The questionnaires for the interviews were also designed and tested. All protocols were developed in alignment with the scientific integrity of exposure assessment, including quality assurance, quality control, and statistical analysis plans. Laboratory analysis of biological samples collected will be conducted by the RSC (e.g., biomarkers of kidney function), and laboratory analyses for biomarkers of environmental exposures will be conducted by designated HHEAR laboratories. Final decisions about exposure assessments methods were made in consideration of logistics, cost, local infrastructure, community acceptance, and the requirement to return results to their participants and the communities as appropriate.

## 4. Questionnaires

The instruments for CURE, administered by trained interviewers, collect comprehensive information at baseline and follow-up visits on demographics, education and household characteristics (National Center for Health Statistics, 2004), family history of kidney disease (Feldman et al., 2024), residential history, drinking water sources, medication including traditional medicinal agents, supplements and herbal remedy use (Quandt et al., 2009), healthcare access (Centers for Disease Control and Prevention, 2015), lifestyle and diet (National Institute of Health, 2024; World Health Organization, 2018; Riley et al., 2016; Global Adult Tabacco Survey Collaborative Group, 2020), and hydration practices. Additional questionnaires were created to assess heat exposure, symptoms related to heat stress and dehydration, occupational exposures (including types of work, tasks, duration, workload, heat exposure, and hydration at work) (Gonzalez-Quiroz et al., 2024; Keogh et al., 2022; Vlahos et al., 2019), and agrochemical exposure at work and at home (Alavanja et al., 1996). Most instruments drew on examples from existing global programs (Table 1) or past CKDu studies, and were adapted for use in CURE. These instruments are implemented using REDCap, with translations in Spanish (for Central American sites) and Telugu (for the site in Andhra Pradesh, India), for baseline and follow-up visits spanning over 2 years.

### 4.1. Occupational exposures, including heat

An occupational questionnaire was designed to collect comprehensive baseline and longitudinal data across a spectrum of employment-related factors, allowing a nuanced characterization of occupational exposures over time and their potential health impacts. This questionnaire built upon previous studies of CKDu in the context of occupational exposures (Gonzalez-Quiroz et al., 2024; Keogh et al., 2022; Vlahos et al., 2019), eliciting details of each participant’s current employment and occupational history. Capturing details of both work history and current employment (repeated longitudinally to capture changes) will allow us to analyze health effects associated with this work both retrospectively and prospectively. The questionnaire captures information about the participant’s primary and secondary sectors of employment (e. g., sugarcane farming, cashew farming, mining, construction, etc.), tasks they complete within those sectors (e.g. harvesting, seeding, watering, weeding, drilling, roofing, etc.), and number of months, days, and hours per week worked (Gonzalez-Quiroz et al., 2024; Keogh et al., 2022; Vlahos et al., 2019).

The questionnaire also captures information on working conditions, including number and duration of breaks taken during the workday, specific conditions under which these breaks occur (e.g., rest in shaded or cooler environments), percentage of manual labor involving physical exertion, the number of days and hours per day spent in outdoor activities each week, amount of time spent working in the sun, and the participant’s experience with exhaustion and sweating related to work. Questionnaires also collect information on the frequency of heat-related illness symptoms such as dizziness, headaches, nausea, muscle cramps, dysuria (Crowe et al., 2015), and hydration practices (including intake of any type of liquids) (Gonzalez-Quiroz et al., 2024; Keogh et al., 2022).

The information collected will allow for estimation of the intensity and duration of occupational exposures, including work conducted in hot environments, which is particularly relevant in regions where heat stress and work-related factors have been proposed as potential risk factors of CKDu.

### 4.2. Pesticide exposures

A questionnaire was developed to gather detailed information on pesticide exposures, modeled in part after the Agricultural Health Study, a prospective cohort study of licensed pesticide applicators from agricultural communities of North Carolina and Iowa, United States (Alavanja et al., 1996). This tool captures a broad range of data, including: the use of specific pesticides at home and in the workplace; involvement in mixing, applying, or loading pesticides; personal experience with repairing pesticide application equipment; types of crops to which pesticides are applied; and the use of personal protective equipment. It also documents any symptoms of pesticide poisoning, a history of pesticide use, and the storage practices for both pesticides and application equipment. The primary list of pesticides of interest was developed by polling all research site principal investigators (PIs) about the most commonly used pesticides in the study regions and consolidating those findings into a 10-item list, to optimize quantitative statistical analyses, with an option for the participant to specify other pesticides used. A catalog with images of the most widely used pesticides in the study communities is shown to participants during the interview to facilitate accurate recall and ensure correct identification of common pesticides.

### 4.3. Behavior and medications

Detailed lifestyle and medications questionnaires were developed based on National Health and Nutrition Examination Surveys (NHANES) and the World Health Organization (WHO) Demographics STEPwise approach (Riley et al., 2016) to assess a range of lifestyle factors, including tobacco use (lifetime use, use within the past 30 days, and age of initiation) (Global Adult Tabacco Survey Collaborative Group, 2020), alcohol consumption over the past 12 months (detailing the types of alcohol) (World Health Organization, 2018), use of products for skin whitening, diet (pork, beef, fish, seafood, rice products, and corn-based products, including the frequency of consumption) (National Institute of Health, 2024), use of plant-based teas, naturopathic remedies, traditional medicines, vitamins, other supplements (recording the frequency of use) and nonsteroidal anti-inflammatory drugs (NSAIDs; recording the frequency of use and dosage) (Quandt et al., 2009). A visual catalog with the NSAIDs most common to each site is shown to participants to facilitate the correct identification of the medicines and estimation of the dosage.

### 4.4. Water source

All participants complete a comprehensive water questionnaire for detailed information on the primary and secondary source of drinking water in the home (e.g., piped water, tube well, dug well, spring and other), the name of water supplier if appropriate and available, whether the source is the same for drinking and cooking, whether the water has undergone treatment such as filtration, chlorination, or boiling, whether water is stored in the home and the types of containers used for storage. These questions are asked for the current season and the opposite season (dry or rainy season) if there are changes. Additionally, for households that provided water samples, the instrument includes detailed documentation of the actual source of the water sample collected and the procedures used for sample collection.

## 5. Biological sample collection and processing

**Fig. 2.**
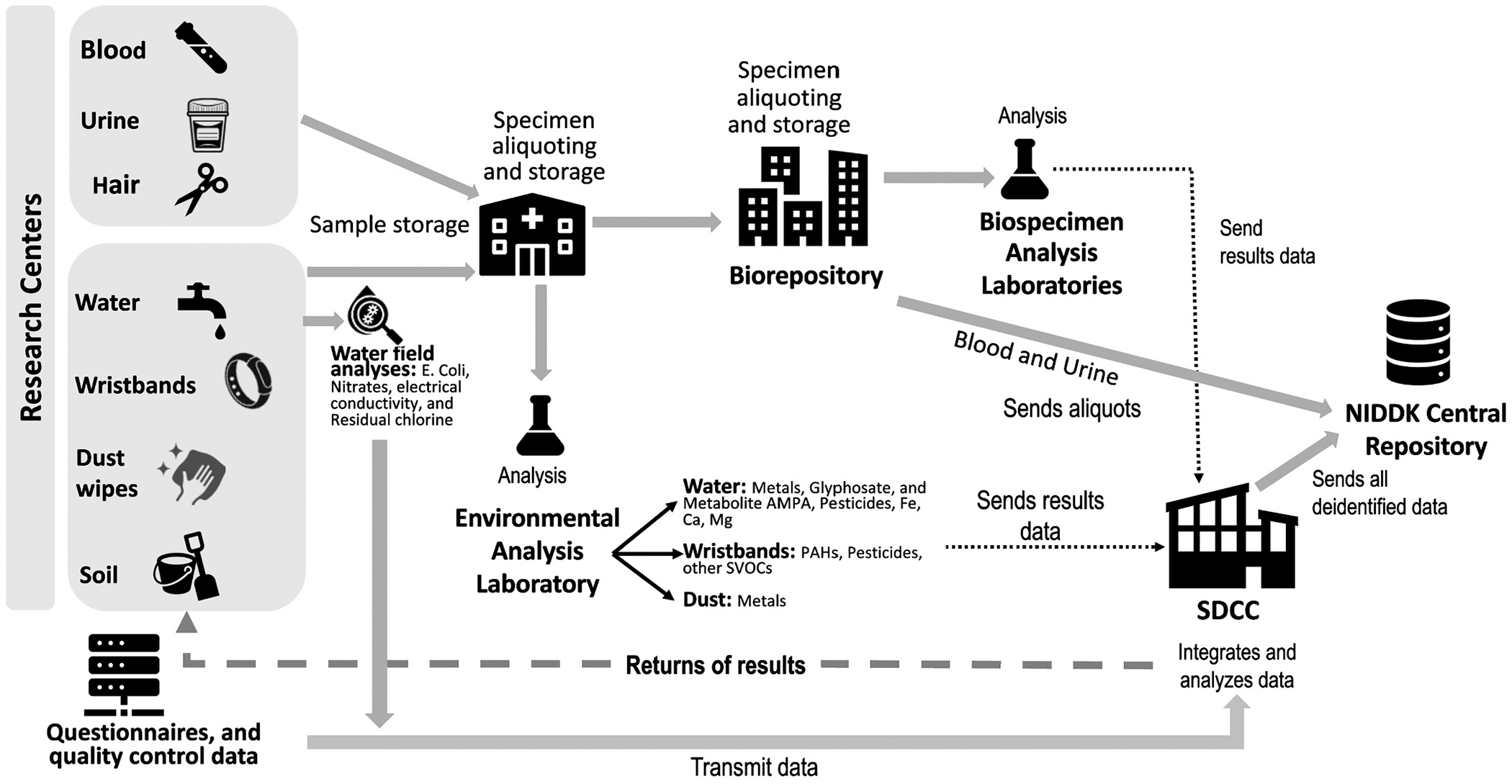
Sample collection and processing operations for environmental exposure assessment.

Sample collection and processing operations for exposure assessment are summarized in Fig. 2. Blood and urine samples are collected at each study visit, and a sample of hair is collected at two time points during the study. All specimen collection materials, such as vacutainers, are provided and pre-labeled by the SDCC and shipped to all sites to maintain standardization across sites. The time of collection and field-storage temperature are recorded electronically in REDCap, with data reviewed by the SDCC. All samples are identified using barcoded labels and are processed, aliquoted, stored locally at −80 °C or the appropriate temperature, and later shipped to the Kryosphere biorepository in the U.S. for further aliquoting and distribution, as needed, to laboratories performing specific analyses. The specific processing and storage steps for each sample type was designed in consultation with the HHEAR laboratories. Details for the collection and processing of each sample type follow the Biospecimens Standardized Operating Procedures (SOP), summarized in Appendix 1.

## 6. Environmental sample collection and processing

The environmental sampling plan aims to provide descriptive information on exposure levels across different sites and to correlate toxicant exposures measured in water, dust wipes, and soil. Silicone wristbands are collected to assess personal exposures resulting from inhalation and dermal absorption of semi-volatile organic chemicals, with an emphasis on pesticides and combustion by-products. Drinking water samples are collected to assess water quality and potential contaminants. Water samples will be tested locally for *E. coli*, residual chlorine, nitrates and electrical conductivity (a proxy for total dissolved solids). Subsequently, samples will be analyzed at a central lab for trace elements and anions such as fluoride, bromide, etc. Dust wipes samples are collected to evaluate environmental exposures from household surfaces. Finally, soil samples are collected to examine the presence of environmental pollutants in the surrounding housed area.

Questionnaire data indicate whether water is supplied from a centralized or municipal source, which informed the selection of a sub-sample of 360 participants for environmental sample collection, including drinking water, dust wipes and silicone wristbands. Environmental sampling takes place once each during the dry season and once during the rainy season (Table 2). The sampling is conducted at the homes of 60 participants per country, equally distributed across the three eGFR categories defined a priori by the CURE Consortium: 20–59, 60–89, ≥90 mL/min/1.73m^2^, and selected randomly within each eGFR category. The information about the participants selected for environmental sampling will be sent to the research site PI and the environmental field team will coordinate with the participants to schedule the sampling visit to their households.

The water samples are collected from the source used for drinking water available in the household, which may be a community water system, a private well, a storage tank, or another source. The wristbands are worn for seven consecutive days by each participant. Dust wipes are collected from a frequently used room on a rarely cleaned horizontal surface where dust accumulates. The soil samples, which are exploratory, are collected from a smaller number of households. Specific processing and storage steps for each environmental sample type were developed in consultation with the HHEAR laboratories. Details of the Environmental Sampling SOP are summarized in Appendix 1.

Global positioning system (GPS) coordinates for residences with environmental samples are captured and preserved locally by individual sites using SurveyCTO^™^. Reporting of results of future geospatial analyses that use these geocoordinates will follow local requirements while protecting participant confidentiality. We expect data from samples collected from these participants will be informative for exposures at a community or other aggregate level, including participants within the same geographic area.

## 7. Field team and staff training

In each country, the FES is comprised of principal investigators, coinvestigators, a field coordinator, interviewers, phlebotomists, lab technicians, and other collaborators, with oversight by and in close communication with the SDCC. One staff member at each FES is designated as the environmental specialist. The SDCC provides the FES staff with the Standard Operating Procedures (SOPs) for clinical visits, biological sampling, and training on the adequate use of REDCap, the platform used for capturing questionnaire responses and data collection (Lebov et al., 2025). Additional in-person training for collecting environmental samples is provided by CURE investigators with environmental sampling expertise and experience in low- and middle-income countries (A. van Geen from the U.S. and C. Ruepert from Costa Rica). The training workshop includes an overview of the contaminants to be measured (pesticides, metals, and infectious agents), a discussion about sociocultural considerations, and a review of effective sample collection practices, local transportation planning, local lab processing and storage procedures, and quality control, per standard operating procedures. Hands-on training enables staff to practice sampling and/or testing water, collecting dust wipes and soil, and deploying wristbands in a consistent manner.

To avoid sample contamination, staff collecting environmental samples are asked not to wear deodorant, perfume, or insect repellant with pyrethroids. If staff members wear insect repellent, sunscreen, or any other product, they are advised to wear the same brand and version at each collection and are required to document this information to allow it to be considered in laboratory and statistical analyses.

## 8. Laboratory analyses

**Fig. 3.**
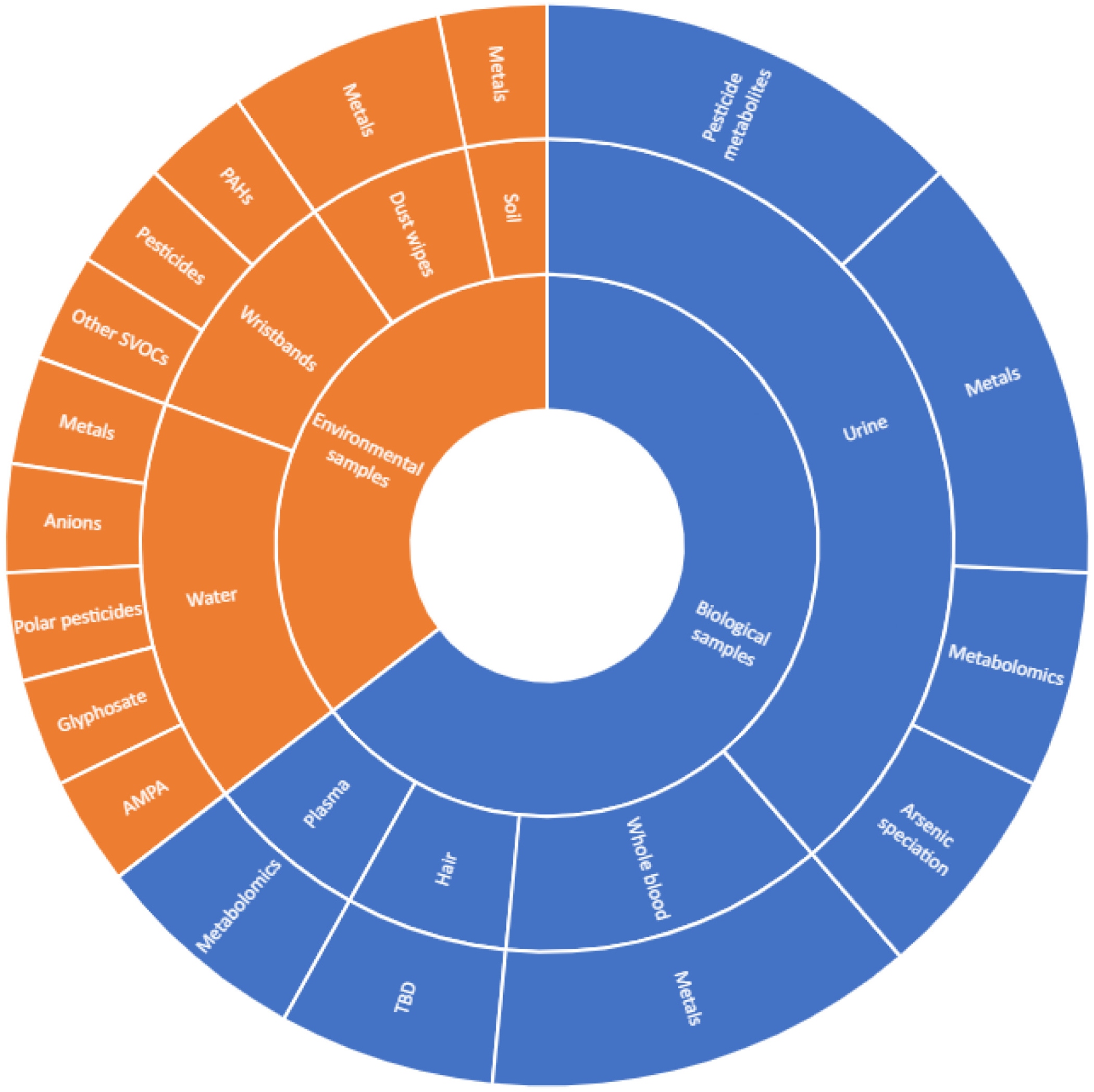
CURE study targeted and untargeted analyses in HHEAR laboratories. TBD: To be determined; samples collected to enable discovery science; several analyses in mind, not yet finalized. Analyses are not exhaustive. Additional volume for ancillary discovery science is available for plasma, urine, hair, whole blood, soil, and wristbands. Metals includes full panel of trace elements (metals and metalloids).

The Human Health Exposure Analysis Resource (HHEAR) laboratories participating in the CURE Consortium and study (Wadsworth HHEAR Laboratory, Minnesota HHEAR Laboratory, North Carolina HHEAR Laboratory at UNC Chapel Hill, and Duke University Environmental Analysis Laboratory) will complete analyses for environmental exposure assessment in biological and environmental samples (Fig. 3). This includes the measurement of trace elements (metals/metalloids), anions, glyphosate, polar pesticides, aminomethylphosphonic acid (AMPA), brominated flame retardants, dioxins, furans, organophosphates (OPEs), polycyclic aromatic hydrocarbons (PAHs), and other chemicals using targeted and untargeted analytical methods (Table 3). Analyses conducted by the HHEAR laboratory network adhere to stringent HHEAR protocols for quality assurance and quality control (QA/QC), as previously described (Ulrich and Ferguson, 2021; Galusha et al., 2021; Kannan et al., 2021).

## 9. Quality assurance and quality control

The CURE Consortium has instituted a wide variety of QA/QC measures to ensure the validity of data generated from biological and environmental samples and from study questionnaires and examinations. All study personnel undergo comprehensive training covering communication, principles of good clinical practice, study implementation and procedures, data entry and verification, and sample collection and handling. A “train-the-trainer” approach is utilized to ensure ongoing skill competency across staff and over the study period. This includes both biological and environmental sample collection to ensure a consistent implementation of the protocol across all sites.

A chain of custody is maintained by requiring staff member identification (written signature) following each collection, processing, and transportation from point of collection to point of final analysis to ensure that specimens are processed and stored within a predetermined acceptable time frame. Shipments of laboratory specimens from the research sites to the United States are tracked, including temperature monitoring, to ensure biospecimens and other samples are well maintained during transit.

## 10. Ethical considerations and informed consent

Prior to enrollment, all participants provide informed consent, ensuring they understand and agree to the study procedures. They are informed of the details and frequency of study visits, sample collection, and safety measures implemented to safeguard their well-being and privacy (including data protection) throughout the process and of potential risks or discomforts. Participation is entirely voluntary, and participants may withdraw at any time during the study. All aspects of the protocol are approved by the single institutional review board (IRB) overseeing U.S. institutions in the consortium along with country and site-specific local IRBs.

## 11. Return of results

The CURE Consortium is committed to engaging with the study participants and their communities about the results of environmental exposure testing. The consortium will return results of locally measured and clinically relevant tests to each participant (blood pressure, body mass index, serum creatinine, glycosylated hemoglobin A1c, and complete blood count once results are available), accompanied by a referral to local clinical care, as appropriate (Lebov et al., 2025). The CURE Consortium has also developed a water result template for field measurements for public and private wells tested for *E. coli*, residual chlorine, nitrates and electrical conductivity (a proxy for total dissolved solids) which will be made available to affected study participants, indicating clearly when results are in exceedance of local and/or WHO guidelines (>0 CFU/100 mL *E. coli*, >5 mg/L residual chlorine, >10 mg/L nitrate as nitrogen) (Appendix 3). The water return-of-results materials were developed through extensive discussion, consensus building, and leveraging local and international experiences, which resulted in a general template with examples that each field site can adapt to the local needs while also considering any regulatory requirements and other community-level considerations. In addition, participants will receive results for other anions, including fluoride and bromide, as well as metals and metalloids. When levels of inorganic chemical contaminants exceed local standards or WHO drinking water guidelines (e.g., arsenic >10 μg/L, lead >10 μg/L, uranium >30 μg/L, fluoride >1.5 mg/L), participants will be informed accordingly (WHO Guidelines Approved by the Guidelines Review Committee, 2017; United States Environmental Protection Agency, 2022). Each site will provide individual test results and reference values/ranges and information on potential associated health risks and recommendations for reducing and preventing future exposures which are developed in collaboration with local stakeholders and official institutions. Each site will be encouraged to recommend follow-up analyses at a local laboratory via appropriate authorities for additional monitoring and reporting for these contaminants.

Metal concentrations in urine and blood are expected to be delivered to the SDCC within six months of the sample being received by the US-certified clinical laboratory. Participants will receive their individual test results for blood lead, cadmium, mercury, and other metals, if their values exceed a predetermined threshold, as confirmed by the HHEAR laboratory under its clinical laboratory permit. This threshold may vary by site based on local requirements or what is deemed actionable by government and health officials. In the absence of local or national thresholds, the following reference ranges will be adopted: blood lead ≥5 μg/dL; blood mercury ≥5 μg/L; blood cadmium ≥5 μg/L. (World Health Organization, 2021) The local researchers will oversee the reporting of individual test results, reference values/ranges, information about health risks, potential exposure sources, and recommendations for reducing and preventing future exposures. The local investigators will determine what is clinically actionable according to local procedures and make appropriate referrals for care. Each site will be encouraged to recommend follow-up analyses at a local clinical laboratory, in consultation with the relevant local authorities, for additional monitoring and reporting of these three blood metals. Aggregate results of blood mercury, blood cadmium and blood lead will be prepared for sharing with local ministries of health and, where appropriate, other key interested parties.

Urine pesticide concentrations are expected to be delivered to the SDCC within six months of the sample being received by the US-certified laboratory. Since there are no global guidelines or EPA standards for urinary pesticide concentrations, the CURE Investigators will review the individual urinary pesticide results and provide recommendations for reporting back. The SDCC will then prepare a summary report, without personal identifiers, for each FES. Local investigators can choose whether and how to share the findings with the community and/or government officials, considering government and IRB expectations while also being cognizant of the political and other implications of sharing these results broadly.

Aggregate results of centralized testing of water, soil, dust wipes, wristbands, and biological samples will be shared with the broader community, as appropriate for each FES, based on community expectations, local policy, or other local considerations. Returned results will, at a minimum, include information on the contaminants detected, the distribution of levels across the community, and potential methods for mitigating subsequent exposure (e.g., using filters or identifying alternative sources for drinking water).

## 12. Data analysis

The CURE study’s primary goal is to evaluate relevant exposures including environmental exposures within the CURE Consortium to characterize (i) exposure levels of the relevant measures in biological and environmental samples (e.g., pesticides such glyphosate and other polar pesticides and trace elements) across CURE sites and by relevant subgroups, (ii) the relationship between the relevant measures in environmental samples and with the corresponding levels in biological samples, and (iii) the relationship of relevant biological and environmental samples with variation in kidney function at baseline and change over the follow-up period. These analyses will be conducted for the individual analytes as well as collectively. Objectives *ii* and *iii* will be conducted overall; stratified analyses by site will be limited for environmental measures due to the small sample size for environmental samples within each country. Beyond environmental and biological samples, similar objectives can be developed for exposures measured through questionnaires. We recognize the potential for identifying spurious associations due to the large number of risk factors being investigated. In our power calculations (see below), we have considered the methods to account for multiple comparisons. All associations will be scrutinized for biologic plausibility and whether related risk factors show similar associations.

Some exposures assessed in the CURE study may have distinct effects on the development/initiation of CKDu versus its progression. We will evaluate associations based on different approaches: (i) sub-populations categorized by baseline kidney function (e.g., eGFR 20–59, 60–89, ≥90 mL/min/1.73m^2^) and (ii) individuals classified as having slow and rapid kidney function decline, based on the slope of kidney function over the longitudinal follow up period. The analyses of associations will include a core geo-demographic variables such as (but not limited to) geographic region (India vs Central America), age, and sex as potential confounders. Any additional confounders required in specific analyses will be identified a priori through a literature review and the development of directed acyclic graphs, ensuring a robust and unbiased assessment of exposure-disease relationships.

Some environmental measures, such as metal(loid)s, pesticides, and other factors, may be highly correlated within chemical groupings or categories, making it difficult to evaluate them simultaneously using traditional regression models. We will apply statistical models that consider mixtures or multiple exposures in relation to outcomes (e.g., multiple pesticides measured in urine) leveraging supervised and unsupervised approaches to account for correlation or high dimensionality. Additionally, we will employ untargeted metabolomics analysis to explore how exposures disrupt endogenous metabolism and their correlations with CKDu, contributing to a deeper mechanistic understanding of the disease.

## 13. Sample size calculations

For environmental exposures, the CURE study was primarily powered to detect differences in mean change in eGFR over time between exposed and unexposed groups. Since eGFR is measured regularly throughout the study, we calculated power by using linear mixed models (LMM) to account for the within-person correlation of multiple measurements over time and adjusting for potential confounding. Additionally, we assumed a standard deviation of 11.4 mL/min/1.73m^2^ (for the difference in eGFR), a within-person correlation between visits of 0.8, a type I error rate of 0.05, and 15 % dropout of participants over time. Since exposure prevalence has an impact on power, we considered a range from 0.1 to 0.4. With our sample size of 3600, a binary (yes/no) exposure, and a single test, we can detect a difference of 2.2, 1.5, or 1.4 mL/min/1.73m^2^ if the exposure prevalence is 0.1, 0.24, or 0.4 respectively. Controlling for multiplicity using a Bonferroni adjustment for 50 tests, we can detect a difference in average change in eGFR of 3.1, 2.2, and 1.9 mL/min/1.73m^2^ (corresponding to the exposure levels). For analyses that are performed within an eGFR category, we can detect a difference of 3.9, 2.9, and 2.6 mL/min/1.73m^2^ (or 5.4, 3.8, and 3.3 mL/min/1.73m^2^ when adjusting for multiplicity) with 1200 enrolled participants. For environmental exposures that are measured only on a subset of participants, the goals were to obtain information of variability within and across sites, variability between seasons, and associations with exposure biomarkers, to inform power analyses for future exposure assessment and association with study outcomes.

## 14. Challenges and considerations

Exposure assessment in the study of CKDu, including environmental assessment, is particularly challenging due to the multifaceted and poorly understood disease etiology. One of the primary challenges is to identify and isolate specific environmental factors that contribute to CKDu among numerous potential sources and exposures. These may include pesticides, metal(loid)s, water quality, work conditions including prolonged and repeated physical exertion, and heat. These exposures require distinct and often complex methodologies for accurate measurement and analysis. Lifestyle (tobacco, alcohol, spices, herbal remedies, other), nutrition, and pharmaceutical exposures are additional relevant factors that are challenging to measure and may present cumulative effects and/or interactions with other risk factors. While previous studies lacked standardized protocols for environmental sampling across regions affected by CKDu, the CURE study is implementing a standardized protocol across six countries to facilitate harmonization and comparison of data across geographic locations. While our repeated environmental sampling approach will allow us to assess variations over seasons and space for a subset of 10 % of cohort participants, these results may indicate the need for more extensive temporal and spatial coverage.

Another challenge is integrating of environmental data with health outcomes to establish causal relationships. Environmental, lifestyle, nutrition, and pharmaceutical exposures can also be considered alongside potential genetic susceptibility, requiring sophisticated analytical approaches and sufficient sample size to disentangle these complex interactions. We anticipate that future work may include genetic analyses in collected biological samples, to investigate potential gene-environment interactions, but this is beyond the current exposure assessment protocol presented here. Additionally, environmental assessments relying on proxies or indirect measures of exposure, such as the concentration of contaminants in dust wipes or water, may be insufficient to capture individual exposure levels. Biological markers of exposures, on the other hand, can represent the body burden and metabolism of multiple contaminants, but not the relevant source(s), and may miss transient or rapidly excreted chemicals. A strength of the CURE study is the exposure measurement in environmental and biological samples as well as questionnaire-based estimation, enabling an in-depth and holistic exposure assessment strategy. Untargeted metabolomics and other molecular techniques can also provide the opportunity to identify new exposures and relevant pathways.

Finally, conducting research in resource-constrained settings requires navigating a complex landscape shaped by political dynamics, infrastructure limitations such as inconsistent electricity, and the distance from the site of assessment or sample collection to local laboratories, especially in rural, relatively impoverished communities. Ensuring the accuracy and reliability of environmental data also requires advanced laboratory facilities and expertise, which are limited in areas where CKDu is prevalent. These factors present significant challenges, yet we have strived to minimize their impacts within our operational planning and research design to ensure a feasible common protocol, with a robust logistical framework for reliable data collection and analysis.

## 15. Conclusions

Rigorous exposure assessment is crucial for elucidating the environmental determinants of CKDu development and progression, as well as appropriately assessing potential gene-environment interactions, which remain poorly understood. Identifying specific contaminants and understanding their pathways of exposure can contribute to the development of effective prevention and intervention strategies. The prospective longitudinal design of CURE, covering multiple timepoints and seasons and multifaceted sample/data collection, will allow us to systematically assess environmental exposures and their potential role in CKDu pathogenesis. The biological and environmental samples, together with extensive questionnaire data, all collected serially, will also facilitate future research and the investigation of novel hypotheses. The overall goal is that this work leads to targeted public health policies and improved health outcomes for populations affected by CKDu.

## Supplementary Material

Supplementary materials

Supplementary data to this article can be found online at https://doi.org/10.1016/j.scitotenv.2025.179642.

## Figures and Tables

**Table 1:** Questionnaires/instruments for exposure characterization.

Exposure category^1^	Instrument [adapted from]	Reference
Demographics, Education and Household Data	United Nations Educational, Scientific and Cultural Organization (UNESCO)	https://uis.unesco.org/UISQuestionnaires/Pages/country.aspx
	Equity tool	https://www.equitytool.org/dhis2/
Residential history and Water Sources	World Health Organization (WHO) United Nations International Children’s Emergency Fund (UNICEF) Joint Monitoring Program (JMP) 2018 Core Questionnaire WHO Joint Monitoring Programme for Water Supply	https://washdata.org/monitoring/methods/core-questions
Current occupation and occupational history	Nicaragua Community-based Cohort	https://bmcnephrol.biomedcentral.com/articles/10.1186/s12882-016-0422-4
	Kidney Protection Project	https://pubmed.ncbi.nlm.nih.gov/30095037/
Pesticide use	Agricultural Health Study	https://www.nature.com/articles/7500232
	Nicaragua Community-based Cohort	https://bmcnephrol.biomedcentral.com/articles/10.1186/s12882-016-0422-4
Alcohol	WHO Global Report	https://www.who.int/publications/i/item/9789241565639
Tobacco	WHO Steps Survey	https://www.who.int/teams/noncommunicable-diseases/surveillance/systems-tools/global-adult-tobacco-survey/questionnaire
Food Frequency	National Cancer Institute Diet History Questionnaire (DHQ) II	https://epi.grants.cancer.gov/dhq2/forms/
Healthcare Access	Centers for Disease Control and Prevention (CDC) Behavioral Risk Factor Surveillance System (BRSS)	https://www.cdc.gov/brfss/index.html
	National Institute of Diabetes AND Digestive and Kidney Diseases (NIDDK) Chronic Renal Insufficiency Cohort (CRIC)	https://repository.niddk.nih.gov/studies/cric/?query=None
	National Institute of Diabetes AND Digestive and Kidney Diseases (NIDDK) Chronic Renal Insufficiency Cohort (CRIC)	https://repository.niddk.nih.gov/studies/cric/?query=None
Kidney Care History	National Institute of Diabetes AND Digestive and Kidney Diseases (NIDDK) Chronic Renal Insufficiency Cohort (CRIC)	https://repository.niddk.nih.gov/studies/cric/?query=None
Alternative Medicines	International Complementary and Alternative Medicine Questionnaire (iCAMQ)	https://pubmed.ncbi.nlm.nih.gov/19388855/
NSAIDs	Drug Dose Equivalent Methods	https://atcddd.fhi.no/ddd/definition_and_general_considera/

1 Instruments used for screening and eligibility are not included.

**Table 2:** Description of environmental samples and timeline by research center.

Research centers	Environmental samples				Timeline	
	Water	Dust	Silicone wristbands	Soil*	Dry season	Rainy season
El Salvador	60	60	60	9	December–April	June–October
Nicaragua	60	60	60	9		
Costa Rica	60	60	60	9		
Guatemala	60	60	60	9		
Panamá	60	60	60	9		
India	60	60	60	9	December–April	June–October
**Total**	**360**	**360**	**360**	**54**		

* Three samples per household and 3 households per country.

**Table 3:** Laboratory analyses for environmental exposure assessment.

Laboratory	Samples^a^	Assay(s)	Measurements^c^
Field tests (on site)	Water	Aquagenx^®^^b^ CHEMetrics^®^ kit CHEMetrics^®^ kit CHEMetrics^®^ kit Traceable conductivity meter pen and standard^e^	*E. coli* Nitrate as nitrogen Residual chlorine Total and soluble iron Electrical conductivity
Wadsworth HHEAR	Whole blood^d^	ICP-MS/MS	Trace elements
	Urine^d^		
	Urine	HPLC-ICPMS/MS	Arsenic speciation
Minnesota HHEAR	Urine	HPLC-MS/MS	Pesticide metabolites
North Carolina HHEAR (RTI/UNC)	Plasma^d^ Urine	UHPLC-HRMS	Metabolomics
Duke HHEAR	Water	ICP-MS Ion chromatography HPLC-MS/MS HPLC-MS/MS	Trace elements Anions Glyphosate, AMPA Polar Pesticides
	Dust wipes	ICP-MS	Trace elements
	Wristbands^d^	GC-EI/GC-ECNI MS	Pesticides, PAHs, other SVOCs
	Soil^d^	ICP-MS	Trace elements

HHEAR: Human Health Exposure Analysis Resource Laboratories ((HHEAR) participating in the CURE Consortium include Wadsworth HHEAR Laboratory, Minnesota HHEAR Laboratory, North Carolina HHEAR Laboratory, and Duke University Environmental Analysis Laboratory.

a Measurements planned for current project period of CURE. Additional laboratory analyses may be possible for samples where the volume and collection strategies enable future ancillary work (e.g., blood, urine, wristbands, water, dust, and soil).

b Aquagenx: Compartment Bag Test *E. coli* + total coliform most-probable number (CBT EC + TC MPN) kits; CHEMetrics^®^ Nitrate Test Kit – Zinc Reduction Method (K-6905), CHEMetrics^®^ Chlorine (Free & Total) Test Kit (K-2504), CHEMetrics^®^ Iron (Total and Soluble) Test Kit (K-6010); GC-EI/ENI: Gas chromatography (GC)/electron capture ionization (EI) or negative ionization (ECNI) mass spectrometry (MS); HPLC: High performance liquid chromatography (HPLC); ICP-MS: Inductively coupled plasma mass spectrometry; MS/MS: tandem mass spectrometry; UHPLC-HRMS: Ultra high-performance liquid chromatography-high resolution mass spectrometry.

c Targeted (urinary) parent pesticides (and corresponding pesticide metabolites): AMPA: Aminomethylphosphonic acid; Diazinon (2-isopropyl-4-methyl-pyrimidinol), Methyl parathion, Parathion (para-Nitrophenol), Chlorpyrifos, Chlorpyrifosmethyl (3,5,6-tricholor-2-pyridinol), 2,4-dichlorophenoxyacetic acid (2,4-dicholorphenoxyacetic acid), Permethrin, Cypermethrin, and Deltamethrin (3-phenoxybenzoic acid), Cyfluthrin (4-fluoro-3-phenoxy-benzoic acid), Permethrin, Cypermethrin, Cyfluthrin (trans-dichlorovinyl-dimethylcyclopropane carboxylic acid), Permethrin, Cypermethrin, Cyfluthrin (cis-dichlorovinyl-dimethylcyclopropane carboxylic acid), Imidacloprid (5-hydroxy-imidicloprid), Malathion (Malathion Diacid), Acetamiprid, Imidacloprid, Thiacloprid (6-chloronicotinic acid), Clothianidin (Clothianidin), Thiacloprid (Thiacloprid), Dinotefuran (Dinotefuran), Sulfoxaflor (Sulfoxaflor).

Trace elements in whole blood: Cadmium (Cd), Chromium (Cr), Cobalt (Co), Copper (Cu), Lead (Pb), Mercury (Hg), Manganese (Mn), Selenium (Se), Zinc (Zn), Antimony (Sb), Arsenic (As), Barium (Ba), Beryllium (Be), Cesium (Cs), Magnesium (Mg), Molybdenum (Mo), Nickel (Ni), Platinum (Pt), Thallium (Tl), Tin (Sn), Tungsten (W), Uranium (U), Vanadium (V).

Trace elements in urine: Arsenic (As), Antimony (Sb), Barium (Ba), Beryllium (Be), Cadmium (Cd), Cesium (Cs), Chromium (Cr), Cobalt (Co), Lead (Pb), Mercury (Hg), Manganese (Mn), Molybdenum (Mo), Nickel (Ni), Platinum (Pt), Thallium (Tl), Tin (Sn), Tungsten (W), Uranium (U), Copper (Cu), Fluoride (F), Magnesium (Mg), Selenium (Se), Strontium (Sr), Tellurium (Te), Vanadium (V), Zinc (Zn).

Arsenic speciation in urine: arsenobetaine (AsB), arsenocholine (AsC), dimethylarsinic acid (DMA), monomethylarsonic acid (MMA), inorganic arsenic (iAs), i.e., the sum of As^3+^ and As^5+^ measured as As^5+^ after oxidation with hydrogen peroxide.

Trace elements and anions in water, soil, dust wipes: Silver (Ag), Aluminum (Al), Arsenic (As), Barium (Ba), Beryllium (Be), Cadmium (Cd), Cobalt (Co), Chromium (Cr), Cesium (Cs), Copper (Cu), Lithium (Li), Magnesium (Mg), Manganese (Mn), Molybdenum (Mo), Nickel (Ni), Lead (Pb), Antimony (Sb), Selenium (Se), Tin (Sn), Strontium (Sr), Thallium (Tl), Uranium (U), Vanadium (V), Zinc (Zn), Fluoride, Nitrate.

Analytes measured in wristbands (chemical groups): Brominated flame retardants, dioxins, furans, organophosphates (OPEs), polycyclic aromatic hydrocarbons (PAHs), personal care products (Lilial), pesticides, phthalates, polychlorinated biphenyls (PCBs), and some industrial chemicals (alkyphenols).

d Additional laboratory analyses are possible for these samples due to additional stored aliquots and collection materials suitable for other types of assays (blood, urine, wristbands, and soil).

e Electrical conductivity measured with a traceable conductivity meter pen (Fisher S98198) and standard (Cole-Parmer 652248).

## Data Availability

No data was used for the research described in the article.

## HIGHLIGHTS

- Chronic Kidney Disease of UnceRtain Etiology (CKDu) is a leading cause of death and disability among young agricultural workers in Central America and Soth Asia.
- The Chronic Kidney Disease of UnceRtain Etiology (CKDu) in Agricultural Communities (CURE) study is a prospective multi-center cohort investigating the complex etiology of CKDu.
- CURE focuses on environmental exposures in rural hotspot regions across Central America and India.
- The study collects data on multiple risk factors—such as social determinants, occupational conditions, heat stress, hydration practices, agrochemical exposures, nephrotoxic medications, metals and metalloids, and other toxicants.
- The study will generate essential knowledge on CKDu risk factors, guiding future research and informing prevention efforts.
