# Supplementary material for: Environmental exposure assessment in the international prospective study of Chronic Kidney Disease of UnceRtain Etiology (CKDu) in Agricultural Communities (CURE) research consortium: Design and protocol development
Source: Sci Total Environ. Author manuscript; Available in PMC 2026 Jun 11. (PMC12445434; doi:10.1016/j.scitotenv.2025.179642)
Supplement: Supplementary materials [file NIHMS2094136-supplement-Supplementary_materials.docx]

***Environmental Exposure Assessment in the International Prospective Study of Chronic Kidney Disease of UnceRtain Etiology (CKDu) in Agricultural Communities (CURE ) Research Consortium: Design and Protocol Development***

**Authors:**

| **Order number** | **Name** | **Email address** | **ORCiD ID (not required)** | **Institutional Affiliation** | **Conflict of interest** |
| --- | --- | --- | --- | --- | --- |
| 1 | Marvin González-Quiroz (corresponding author) | gonzalezquir@uthscsa.edu | 0000-0002-0093-6357 | Department of Environmental and Occupational Health, UT School of Public Health San Antonio, The University of Texas Health Science Center at San Antonio, San Antonio, Texas, USA  Centre for Kidney and Bladder Health, University College London, London, UK | None |
| 2 | Anna Aceituno | [aaceituno@rti.org](mailto:aaceituno@rti.org) | 0000-0002-2610-521X | RTI International  3040 E. Cornwallis Rd.  Research Triangle Park, NC, US 27709-2194 | None |
| 3 | Shuchi Anand | sanand2@stanford.edu | [0000-0002-9892-8477](http://orcid.org/0000-0002-9892-8477) | Stanford University School of Medicine, Medicine, Division of Nephrology  780 Welch Road  Suite 106  Palo Alto, CA, US 94304  650-725-4738 | None |
| 4 | Alexander van Geen | avangeen@ldeo.columbia.edu | 0000-0003-2073-9841 | Lamont Research Professor, Geochemistry, Lamont-Doherty Earth Observatory (LDEO), Columbia Climate School. New York, NY, USA | None |
| 5 | Lawrence S. Engel | larry.engel@unc.edu | 0000-0001-9268-4830 | University of North Carolina,   Department of Epidemiology, Gillings School of Global Public Health, Chapel Hill, NC  919-962-2756 | None |
| 6 | Emmanuel Jarquin | emmrijarquin@gmail.com | 0009-0003-2942-1909 | Agency for Agricultural Development and Health (AGDYSA)  Col. Las Rosas, P. Las Margaritas 15  San Salvador, San Salvador, SV 01101  2274 7139 | None |
| 7 | Clemens Ruepert | clemens.ruepert@una.cr | 0000-0001-5109-2222 | Universidad Nacional de Costa Rica  Regional Institute for Studies on Toxic Substances,    Heredia, Heredia, CR 86-3000 | None |
| 8 | Nicole Villegas-González | nicole.villegas@ucr.ac.cr | 0000-0003-0006-3872 | Escuela de Tecnologías en Salud, Universidad de Costa Rica, Sede de Guanacaste  Liberia, Costa Rica  315000 | None |
| 9 | Mariela Arias-Hidalgo | mariela.ariashidalgo@ucr.ac.cr | 0000-0001-8979-5409 | Escuela de Medicina, Universidad de Costa Rica  Sede Rodrigo Facio  11501-2060 San José, Costa Rica | None |
| 10 | Nora Franceschini | noraf@unc.edu | [0000-0001-9755-2175](http://orcid.org/0000-0001-9755-2175) | University of North Carolina, Epidemiology, School of Public Health  137 E. Franklin Street, suite 306 CB#8050  Chapel Hill, NC, US 27514-3628  919-966-1949 | None |
| 11 | Daylin Anchía-Pastrán | daylin.anchia.pastran@una.cr |  | Universidad Nacional de Costa Rica  Regional Institute for Studies on Toxic Substances,   Heredia, Heredia, CR 86-3000 | None |
| 12 | Karla Solano-Diaz | karla.solano.diaz@una.cr |  | Universidad Nacional de Costa Rica   Regional Institute for Studies on Toxic Substances,    Heredia, Heredia, CR 86-3000 | None |
| 13 | Andrea Corrales-Vargas | andrea.corrales.vargas@una.cr | 0000-0003-4050-3414 | Universidad Nacional de Costa Rica  Regional Institute for Studies on Toxic Substances,   Heredia, Heredia, CR 86-3000 | None |
| 14 | Jennifer Crowe | jennifer.crowe@una.cr | 0000-0002-0608-7157 | Universidad Nacional de Costa Rica  Regional Institute for Studies on Toxic Substances,   Heredia, Heredia, CR 86-3000 | None |
| 15 | Idalina Cubilla-Batista | [idacubilla@gmail.com](mailto:idacubilla@gmail.com) | 0000-0003-2269-1730 | Hospital Regional Dr. Rafael Estevez  Avenida Alejandro Tapia Final Aguadulce, Coclé, República de Panamá | None |
| 16 | Hildaura Acosta | hildaura.depatino@up.ac.pa | 0000-0001-6574-587X | Universidad de Panamá, Vicerrectoría de Investigación y Postgrado, Facultad de Medicina, Centro de Investigación e Información de Medicamentos y Tóxicos (CIIMET), Panamá, Panamá. | None |
| 17 | Adriana Mike | adriananmc@gmail.com | 0009-0006-0735-4399 | Universidad de Panamá, Centro de Investigación e Información de Medicamentos y Tóxicos (CIIMET), Panamá, Panamá | None |
| 18 | Carolina Guzmán-Quilo | guzmanquilocarolina@gmail.com | 0000-0001-7607-5787 | Universidad de San Carlos de Guatemala  Departamento de Toxicología, Facultad de Ciencias Químicas y Farmacia  3a calle 6-47 zona 1, ciudad de Guatemala 01001 | None |
| 19 | Aurora Aragón | auroraragon@gmail.com | 0000-0003-2216-552X | Wuqu' Kawoq  2 Calle 5-43 Zona 1  Santiago Sacatepéquez, Sacatepéquez, GT 03006 | None |
| 20 | Indiana López-Bonilla | indianalopezb@gmail.com | 0000-0002-0760-1111 | Wuqu' Kawoq  2 Calle 5-43 Zona 1  Santiago Sacatepéquez, Sacatepéquez, GT 03006 | None |
| 21 | Peter Rohloff | [peter@wuqukawoq.org](mailto:peter@wuqukawoq.org) | [0000-0001-7274-8315](http://orcid.org/0000-0001-7274-8315) | Wuqu' Kawoq  2da Avenida 3-48  Barrio Pacatabaj, Tecpán, Chimaltenango , GT 04006 | None |
| 22 | Madeleine K Scammell | mls@bu.edu | 0000-0003-3836-083X | Boston University School of Public Health, Department of Environmental Health  715 Albany St.  Boston, MA, US 02118 | None |
| 23 | Ramón Garcia-Trabanino | rgt@anhaes.org | 0000-0002-4196-9585 | Agency for Agricultural Development and Health (AGDYSA)  Col. Las Rosas, P. Las Margaritas 15  San Salvador, San Salvador, SV 01101  2274 7139  Centro de Hemodiálisis,  C. Gabriela Mistral 211  San Salvador, San Salvador, SV 01101  22254431 | None |
| 24 | Juan Amador Velázquez | juanjoseamador3011@gmail.com |  | Boston University School of Public Health, Department of Epidemiology  Boston, MA, US | None |
| 25 | Daniel R Brooks | danbrook@bu.edu | 0000-0001-6220-6889 | Boston University  715 Albany Street  Department of Epidemiology  Boston, MA, US 02215-1300 | None |
| 26 | Sumit Mohan | sm2206@cumc.columbia.edu | [0000-0002-5305-9685](http://orcid.org/0000-0002-5305-9685) | Columbia University Medical Center, Division of Nephrology, Department of Medicine,  and Department of Epidemiology, 622 W 168th St PH4-124  New York, NY, US 10032 | None |
| 27 | Jai Radhakrishnan |   jr55@cumc.columbia.edu |  | Columbia University Medical Center, Division of Nephrology, Department of Medicine | None |
| 28 | Balaji Gummidi | bgummidi@georgeinstitute.org.in | 0000-0002-1444-3262 | George Institute for Global Health, UNSW, New Delhi, India  Prasanna School of Public Health, Manipal Academy of Higher Education, Manipal, India | None |
| 29 | Vivekanand Jha | vjha@georgeinstitute.org.in | [0000-0002-8015-9470](http://orcid.org/0000-0002-8015-9470) | George Institute for Global Health, UNSW, New Delhi, India;  School of Public Health, Imperial College, London, UK;  **Prasanna School of Public Health, Manipal Academy of Higher Education, Manipal, India**. | VJ reports consulting fee/honoraria from Bayer, Astra Zeneca, Boehringer Ingelheim, Biocryst, Vera, Visterra, Otsuka, Novartis, Astra Zeneca, Chinook, Biocryst and Alpine under the policy of all payments going to the organization |
| 30 | Whitney Collado | wc2837@cumc.columbia.edu |  | Columbia University Mailman School of Public Health, Department of Environmental Health Sciences  New York, NY, US | None |
| 31 | Vivek Bhalla | vbhalla@stanford.edu | 0000-0002-5420-9657 | Stanford University School of Medicine, Medicine  Division of Nephrology, 3180 Porter Drive, Stanford, CA, US 94305 650-721-2471 | None |
| 32 | David J  Friedman | dfriedma@bidmc.harvard.edu | 0000-0002-0301-9298 | Beth Israel Deaconess Medical Center, Renal Division, 330 Brookline Ave  RN 227B  Boston, MA, US 02215  617-667-0253 | None |
| 33 | Sushrut S Waikar | swaikar@bu.edu | [0000-0003-4004-326X](http://orcid.org/0000-0003-4004-326X) | Section of Nephrology, Boston Medical Center and Boston University Chobanian & Avedisian School of Medicine  650 Albany Street, EBRC5  Boston, MA, US 02118 | None |
| 34 | Karen Kesler | kkesler@rti.org | 0000-0002-4425-7348 | RTI International  P O Box 12194  3040 Cornwallis Rd.  Research Triangle Park, NC 27709 | None |
| 35 | Lillian Trochinski | [ltrochinski@rti.org](mailto:ltrochinski@rti.org) |  | RTI International  3040 E. Cornwallis Rd.  Research Triangle Park, NC, US 27709-2194 | None |
| 36 | P. Lee Ferguson | lee.ferguson@duke.edu |  | Department of Civil and Environmental Engineering, Duke University, Durham, North Carolina 27708, United States | None |
| 37 | Patrick J. Parsons | [patrick.parsons@health.ny.gov](mailto:patrick.parsons@health.ny.gov) | 0000-0001-9133-875X | Division of Environmental Health Sciences, Wadsworth Center, New York State Department of Health, Empire State Plaza, Albany, NY 12237  Department of Environmental Health Sciences  College of Integrated Health Sciences  University at Albany  1 University Pl, Rensselaer, NY 12144 | None |
| 38 | Heileen Hsu-Kim | hsukim@duke.edu | 0000-0003-0675-4308 | Duke University, Department of Civil & Environmental Engineering, Box 90287, Durham, NC 27708 USA | None |
| 39 | Carin Huset | carin.huset@state.mn.us | 0000-0002-4538-9326 | Public Health Laboratory, Minnesota Department of Health, Saint Paul, MN, 55101 USA | None |
| 40 | Susan Summer Jenkins | susan_sumner@unc.edu |  | Department of Nutrition, Gillings School of Global Public Health  Department of Pharmacology, UNC School of Medicine  University of North Carolina at Chapel Hill | None |
| 41 | Susan R. Mendley | susan.mendley@nih.gov | 0000-0001-5032-9136 | National Institute of Diabetes and Digestive and Kidney Diseases, Division of Kidney, Urologic and Hematologic Disease  2 Democracy Plaza  6707 Democracy Blvd  Bethesda, MD, US 20892-5458  3018271861 | None |
| 42 | Jill F. Lebov | jlebov@rti.org | [0000-0003-4992-8363](http://orcid.org/0000-0003-4992-8363) | RTI International  3040 E. Cornwallis Rd.  Research Triangle Park, NC, US 27709-2194 | None |
| 43 | Bonnie R. Joubert | bonnie.joubert@nih.gov | 0000-0001-7952-1180 | National Institute of Environmental Health Sciences, Division of Extramural Research and Training  Durham, NC, US | None |
| 44 | Ana Navas-Acien | an2737@cumc.columbia.edu | 0000-0001-9824-7797 | Columbia University Mailman School of Public Health, Department of Environmental Health Sciences  New York, NY, US | None |

**Corresponding author:** Marvin González-Quiroz.

*Affiliation:* Department of Environmental and Occupational Health, UT School of Public Health San Antonio, The University of Texas Health Science Center at San Antonio, San Antonio, Texas, USA

*Address:* 8403 Floyd Curl Drive | Mail Code 8363 | San Antonio, TX 78229

*Tel:* + (1) 2108573611

*Email:* [gonzalezquir@uthscsa.edu](mailto:gonzalezquir@uthscsa.edu)

## SUPPLEMENTARY MATERIAL

## Appendix 1. Biological Sample Collection Procedures

### Blood

Whole blood, serum, and plasma samples are collected during each visit by trained phlebotomists following a small set of questions about recent food intake. At enrollment visit 1, one vacutainer with polymer clotting gel and silica clot activator (BD 367981) for serum, one with lithium heparin for plasma (BD 366667), and one vacutainer with K_2_EDTA certified for trace elements in whole blood (BD 368381), and one with K_2_EDTA (BD 367856) for local analysis of whole blood for a Complete Blood Count (CBC) with differential are collected using aseptic techniques. The serum and plasma vacutainers with clotting gel and lithium heparin are centrifuged in the field following manufacturer-recommended incubation periods, while the whole blood samples with K_2_EDTA are immediately placed in 2-8°C conditions (refrigerator or cooler with ice packs and temperature monitor).  Each vacutainer is labeled, centrifuged (if applicable), and stored according to the SOP. The serum, heparinized plasma, and trace-element-free whole blood are stored for centralized measurement of kidney function and injury biomarkers, genetics, trace elements, other environmental parameters of interest, and for discovery science. DNA, RNA, and other measures may be available from samples collected at follow-up timepoints.

### Urine

At every study visit, study staff ask participants to provide a spot sample at study visit after providing participants with instructions on proper self-collection of the urine specimen (mid-stream clean catch void). Participants are asked to wash hands with water only and to use only 100% water wipes for cleaning themselves, if necessary, prior to sample collection, to avoid sample contamination with soap products. Each urine specimen cup is labeled, processed and aliquoted to cryovials that contain unspun urine, spun urine, or sediment, which is stored at -80^o^C in a local freezer before being shipped to Kryosphere biorepository in the US.

### Hair

Trained field team members collect the hair samples. First, they put on gloves and disinfect the scissor blades with alcohol wipes, ensuring complete air drying. Then, they identify the vertex posterior of the participant’s scalp by drawing an imaginary line from the tips of the ears toward the back of the head, selecting the site for hair collection along this midline. If the hair is more than 3 cm long, they select approximately 100 strands of hair (around a pencil width), securing tightly with index finger and thumb and tie a 20 cm floss around the hair as proximal to the scalp as possible, cut the hair as close to the scalp over the floss as possible, ensuring secure attachment to the hair root, and place the hair sample into the designated envelope, discarding any surplus hair and used materials. If hair length is <3 cm, the hair is directly deposited into the labeled envelope without using the floss. At the end of hair collection, the scissor blades are cleaned with alcohol wipes. The hair sample is stored at room temperature (15 to 25°C) within the specified storage box, avoiding exposure to smoke, sunlight, or artificial light to maintain sample integrity.^31-33^

## Appendix 2. Environmental Sample Collection Procedures

### Water

For each participant, the environmental sampling team first identifies the primary source of water used for drinking at the residence (tap or well on the property, or storage container/tank if the source is off the property). Water samples are collected from this primary source wearing disposable gloves. For up to 10 households per country, at the discretion of the field team, an additional sample of water is collected to capture the water closest to consumption (e.g., water from a smaller container such as a pitcher, which may have treated prior to storage). Field measurements to assess water quality and reporting results to the participants are a service provided by CURE investigators to local communities. To identify microbial contamination in drinking water and quantify the Maximum Probable Number (MPN) of organisms, the Aquagenx multi-compartment bag incubation system is used. This system detects *E. coli* and is comparable to the IDEXX Colilert QuantiTray 2000 method endorsed by the US EPA (WHO JMP 2022).^34^ The environmental sampling team collects two water samples simultaneously from the same source, treating them identically. *E. coli* levels serve as indicators of microbial contamination in drinking water,^35^ aligning with WHO guidelines for *E. coli* detection.^28^ Additionally, residual chlorine levels are measured using the CHEMets kit for free chlorine to identify potential reasons for any microbial contamination. Electrical conductivity is measured as a proxy for total dissolved solids, using a probe that is calibrated daily against a 1000 uS/cm standard and ultra-pure water. Finally, dissolved iron and nitrate are also measured using CHEMets kits to help distinguish between surface and groundwater, as well as between oxic and anoxic groundwater conditions.

#### Trace elements

Water samples for measuring a panel of 24 metal(loid)s are collected directly, without filtration, into 20 mL scintillation vials to reflect actual participant intake and to minimize contamination from handling. These samples are not acidified until receipt at the HHEAR analytic lab.^29^ Water samples for analysis of anions, such as fluoride and nitrate, are collected, with filtration, into vials in the field. The anion vials are stored at ≤-1°C within 24 hours of collection and maintained at this temperature until analysis. The metal(loid) vials are stored at either 2-8°C or ≤-1°C until shipment to the HHEAR laboratory, where they are stored at ≤-1°C until analysis.

#### Pesticides

Forty milliliters of water are directly collected into 50 mL centrifuge tubes for the analysis of glyphosate and one of its primary degradation products, aminomethylphosphonic acid (AMPA). Immediately after collection, samples are spiked with isotopically-labeled surrogate standards^25,36^ Samples and field blanks are transported on ice to the local laboratory and stored at -20ºC within 24 hrs.

For quality control, duplicate spiked samples are collected from 5% of sources, distributed approximately evenly throughout the sampling period. At every tenth sample location, a field blank is prepared. A field blank consists of a 50 mL centrifuge tube labeled as a field blank, pre-filled with 40 mL of LC-MS grade water at the laboratory on the morning of sampling, capped, and transported with the empty sample collection bottles to the sampling sites. At the sites where blank samples are obtained, the capped field blank tube is removed from the sample cooler along with the empty sample tube intended for water collection. During sample collection, the field blank is uncapped and briefly exposed to the same environment as the sample tube. After collection, both the field blank and the water sample are capped and placed back in the cooler for transport to the local laboratory. Following processing at this laboratory, the water samples for glyphosate analysis and associated field blanks are stored at -20^o^C. The frozen samples are shipped with dry ice to the HHEAR laboratory in the US.

#### Other polar pesticides

Water samples for the analysis of polar pesticides, are collected using a 1L glass bottle rinsed between samples with the best grade of methanol and water available at each site. Ideally, 1L of water is collected directly into the bottle without using a sampler. If a sampler is used, both of the bottles are rinsed 3 times prior to water collection. After collection, the glass bottle and any field blank bottle are stored on ice in a cooler and kept refrigerated and away from light until extraction. The water is processed using Solid-Phase Extraction (SPE) manifold within 24 hrs. Up to six sample bottles are set near the SPE manifold and tubes from each sample bottle are connected to corresponding Oasis HLB Prime cartridges into Visiprep DL valve liners. The cartridges are loaded onto the Visiprep DL soil-phase extraction manifold. The vacuum is started and adjusted to a medium level (10-15 in Hg). The cartridge valves are opened, and the flow rate is set at 1-2 drops per second. For water samples with turbidity above approximately 100 mg/L of suspended solids or if clogging is observed, a filtration step must be applied before the SPE protocol. This method is robust and requires no pre-conditioning (pre-rinsing with solvents and water) of the vacuum cartridges^37^. Isotopically-labeled surrogate standards (stable, not radioactive) prepared in methanol are added to the sample before extraction to account for losses in recovery.^37^ The cartridges are stored at -20^o^C in the local laboratory. During shipment, cartridges are kept cold/frozen on dry ice to minimize temperature fluctuations prior to and during shipping back to the HHEAR laboratory for analysis.

### Dust wipes

For dust wipe collection, the field team selects a room with high activity or in which the participant spends the most time. The team then identifies a large, infrequently cleaned, horizontal surface, such as the top of a refrigerator, cabinet, bookshelf, door frame, dresser, or floor, where dust accumulates. Wearing non-powdered disposable gloves, a 30 x 30 cm square template is placed on the surface. The area is wiped methodically: first from the top right to the top left, then to bottom right, and finally to the bottom left, folding the wipe inward and this is repeated with a new wipe in the opposite direction. Both wipes are placed in 50 mL digestion tubes labeled with the participant ID. Field blank samples are collected from 5% of households, distributed approximately evenly throughout the sampling period by carefully unfolding the wipe without letting it contact any surfaces or dust, then folding it four times and placing it in the 50 mL digestion tube labeled Blank. The samples are transported to the local laboratory on ice and stored at 4°C or colder until shipping to the Duke HHEAR lab on dry ice for analysis of nineteen trace elements.

### Wristbands

Participants are provided with sterile silicone wristbands on the same day that water and dust samples are collected. They are instructed to wear the wristband continuously for seven days to ensure consistent exposure assessment. Participants are instructed not to remove or tamper with the wristbands during this period. Afterward, the wristbands are carefully removed, wrapped in aluminum foil, and sealed in individually labeled bags. Field blanks are collected on the first day of sampling by briefly exposing an unused wristband to the air before sealing it in a labeled package. Wristbands are stored in their bags, protected from light, moisture, and extreme temperatures (ideally at 4^o^C). They are transported to the local laboratory in a cooler at 4^o^C and stored at -20^o^C within 24 hours of removal or at room temperature for up to one week, before being shipped to the corresponding HHEAR laboratory on dry ice for analysis.

### Soil

A total of 9 soil samples are randomly collected from three households in each country (up to 60 residences total across the six countries). Approximately 50 grams of soil are collected from 1-2 centimeters below the surface at 3 locations: the main entrance, the backyard, and the street or road in front of the house, along with a photograph and description of each spot. Samples are collected into amber glass jars, transported to the local lab at room temperature and stored at -1°C or below for subsequent shipping to the corresponding HHEAR laboratory on dry ice in accordance with US Department of Agriculture requirements. The laboratory will analyze a panel of trace elements in soil samples.

## Appendix 3. Return of Results Template for Water Field Measurements

Dear [NAME],

Thank you for participating in this study measuring contaminants in water samples to learn more about drinking water exposures for agricultural communities in [TOWN, COUNTRY]. We are pleased to share information back regarding your results for the water samples collected in your home on [DATE].

Your water sample was analyzed for some properties at your home to assess water quality. In this report we provide the results of these analyses.

Your water samples will also be analyzed in a central laboratory in the United States for pesticides and metals. The results of these analyses take longer and will be returned later.

The attached tables include the complete results for the analyses conducted at your home. We have included guidelines from the World Health Organization for your reference. Country-level guidelines might be different and are provided as well when available. **This is NOT a medical test, and these results are not enough to determine your state of health**.

We have color coded the results of individual contaminants measured in your water sample and provided general recommendations given the contaminant levels measured in your water. The explanation of the colors and the relevant public health recommendations are at the end.

We have included a color at the end to help you understand your water results and relevant public health recommendations:

If you have any questions about these results and recommendations, you can contact us at the following [contact information]

Your tap water results and any relevant public health recommendations are presented here:

**Your water results (Example 1):**

| **Contaminant** | **Your water’s measurement** | **Comparison values (WHO)** | **National/local recommended value** | **Meaning** | **Recommended action** |
| --- | --- | --- | --- | --- | --- |
| E. coli  (cfu/100 mL)* | Sample 1: none | None | [INSERT] | None detected | None |
|  | Sample 2: none |  |  |  |  |
| Residual chlorine** (mg/L) | 0.4 | ≤5  Target: 0.2 to 1.0 | [INSERT] | Levels within limit and target | None |
| Nitrate (mg/L)*** | None | ≤11 | [INSERT] | None detected | None |

cfu: colony forming units

*E. coli was measured in 2 samples

** Note: this is only for chlorinated water

***Expressed as nitrogen

**Your water results (Example 2):**

| **Contaminant** | **Your water’s measurement** | **Comparison values (WHO)** | **National/local recommended value** | **Meaning** | **Recommended action** |
| --- | --- | --- | --- | --- | --- |
| E. coli  (cfu/100 mL)* | Sample 1:  none | None | [INSERT] | Medium risk - E. coli was detected suggesting presence of fecal material in the sample | Monitor and consider treatment |
|  | Sample 2:  5.2 |  |  |  |  |
| Residual chlorine (mg/L)** | 0.1 | ≤5  Target: 0.2 to 1.0 | [INSERT] | Levels below the low limit of target | Consider water treatment |
| Nitrate (mg/L)*** | 0 | ≤11 | [INSERT] | None detected | None recommended |

cfu: colony forming units; WHO: World Health Organization

*E. coli was measured in 2 samples

** Note: this is only for chlorinated water

***Expressed as nitrogen

**Your water results (Example 3):**

| **Contaminant** | **Your water’s measurement** | **Comparison values (WHO)** | **National/local recommended value** | **Meaning** | **Recommended action** |
| --- | --- | --- | --- | --- | --- |
| E. coli  (cfu/100 mL)* | Sample 1:  >100 | None | [INSERT] | Very high risk - E. coli was detected suggesting presence of fecal material in the sample | Switch water sources or add chlorine to water.    If you are using a container to store water, use a covered container with a spigot |
|  | Sample 2:  >100 |  |  |  |  |
| Residual chlorine (mg/L)** | 0.1 | ≤5  Target: 0.2 to 1.0 | [INSERT] | Levels below the low limit of target | Consider water treatment |
| Nitrate (mg/L)*** | 0 | ≤11 | [INSERT] | None detected | None recommended |

cfu: colony forming units; WHO: World Health Organization

*E. coli was measured in 2 samples

** Note: this is only for chlorinated water

***Expressed as nitrogen

**Color key scale to help you interpret your results:**

**E.coli:** The results for *E.coli* contamination levels are shown on a gradual scale for tap water, where each range indicates a different level of health concern. We used the highest value of your two water samples to indicate the color on the scale.

| ***E. coli* contaminant level in your water** | **Recommended action** |
| --- | --- |
| <1 cfu/100 mL | None recommended |
| 1-10 cfu/100 mL | Medium risk – Monitor |
| 10-100 cfu/100 mL | High risk – Consider switching water sources or adding chlorine to water.    If you are using a container to store water, we recommend using a covered container with a spigot. |
| >100 cfu/100 mL | Very high risk – Switch water sources or add chlorine to water    If you are using a container to store water, use a covered container with a spigot. |

**Residual chlorine:** These levels and recommendations only apply if the water is chlorinated

| **Residual chlorine level in your water** | **Recommended action** |
| --- | --- |
| Residual chlorine measured above 5 mg/L [WHO] | Switch water sources or water treatment, if possible |
| Residual chlorine measured below 0.1 mg/L and above 2 mg/L, this level is considered out of target | Consider switching water sources or water treatment |
| Residual chlorine detected between 0.1 mg/L and 2 mg/L is considered within target | None recommended |

**Nitrate**

| **Nitrate level as nitrogen in your water** | **Recommended action** |
| --- | --- |
| Nitrate level measured as nitrogen above 11 mg/L [WHO] | Switch water sources or water treatment, if possible |
| Nitrate level measured as nitrogen ≤11 mg/L | None recommended |

*Please note: This color key is intended to inform recommendations for individual contaminants and does not necessarily consider the mixture of contaminants that could occur in water. If you have multiple contaminants in the yellow or red range, please consider switching water sources. You can contact us so we can help you understand your results.*

**What can I do if my water is contaminated and/or does not meet the recommended values either because the levels are too high or too low?**

| ***Escherichia coli (E. coli)*** | - Identify sources of contamination - If a tank is used to store water, clean the tank. - Chlorinate your tank more frequently. - Boil your water - If possible, switch water sources |
| --- | --- |
| **Residual chlorine** | - If you are using a tank, we recommend cleaning it regularly to prevent contamination and maintain chlorine effectiveness. - We recommend contacting your drinking water operator to add chlorine more frequently. |
| **Nitrate** | - We recommend switching water sources |

**What are my next steps?**

Depending on the contaminants in your water, we either have no recommendations or we recommend switching water sources or treating your water if possible.

Please contact us at the following contact information if you have questions about your results:

Name: ___________

Phone number: ____________

Address: _________________

Here are some resources about water quality, safety, and contaminants in drinking water:

***World Health Organization:*** *The World Health Organization (WHO) produces the Guidelines for drinking-water quality (GDWQ) to protect public health.*

**Guidelines for drinking water-quality (GDWQ):**

- <https://www.who.int/teams/environment-climate-change-and-health/water-sanitation-and-health/water-safety-and-quality/drinking-water-quality-guidelines>


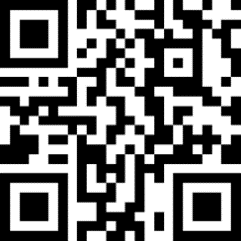


**Water safety planning for small community water supplies:**

- <https://www.who.int/publications/i/item/9789241548427>


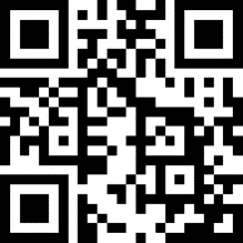


***U.S Environmental Protection Agency (EPA):*** *The Environmental Protection Agency (EPA) establishes and enforces regulations to protect human health and the environment.*

**Water Health Series – Filtration Facts**

- <https://www.epa.gov/sites/default/files/2015-11/documents/2005_11_17_faq_fs_healthseries_filtration.pdf>


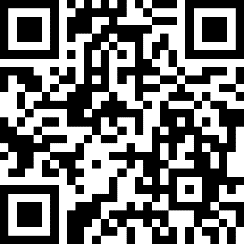


**Factsheet on water quality parameters: E. coli (Escherichia coli)**

- <https://www.epa.gov/system/files/documents/2021-07/parameter-factsheet_e.-coli.pdf>


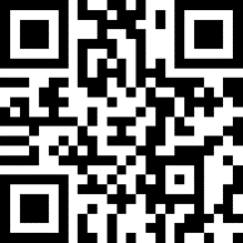


***Agency for Toxic Substances and Disease Registry (ASTDR):*** *The Agency for Toxic Substances and Disease Registry (ATSDR) protects public health by managing hazardous substance exposure.*

**Information on Nitrate**

- <https://wwwn.cdc.gov/TSP/substances/ToxSubstance.aspx?toxid=258>


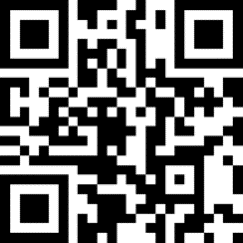


***Centers for Disease Control (CDC):*** *The Centers for Disease Control and Prevention (CDC) safeguards public health through disease prevention, control, and health promotion.*

**Germs that can contaminate tap water:**

- https://www.cdc.gov/drinking-water/causes/germs-that-can-contaminate-tap-water.html?CDC_AAref_Val=https://www.cdc.gov/healthywater/drinking/contamination/germs.html


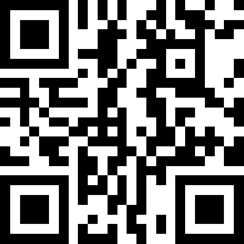


Thank you again for your participation in this study. If you have any questions about these recommendations, you can contact us at the following [contact info]

[SIGNATURE]
